# Supplementary material for: Feeling Stressed and Ugly? Leave the City and Visit Nature! An Experiment on Self- and Other-Perceived Stress and Attractiveness Levels
Source: Int J Environ Res Public Health. 2020 Nov 17;17(22):8519. doi: 10.3390/ijerph17228519 (PMC7698395; doi:10.3390/ijerph17228519)
Supplement: Supplementary file 1 [file ijerph-17-08519-s001.pdf]

Table S1. Correlations between attractiveness and stress ratings.

		Self		Other	
		r	*p*	*r*	*p*
pre	nature	−0.73	<0.001	−0.63	0.002
	urban	−0.54	0.014	−0.61	0.003
post	nature	−0.34	0.147	−0.60	0.004
	urban	−0.18	0.438	−0.17	0.466

Table S2. Correlations between self- and other-ratings.

		Stress		Attractiveness	
		r	*p*	*r*	*p*
pre	nature	0.19	0.432	0.44	0.054
	urban	0.43	0.061	0.28	0.228
post	nature	−0.19	0.419	0.16	0.510
	urban	0.19	0.427	0.24	0.309
